# Gout Flares and Mortality After Sodium-Glucose Cotransporter-2 Inhibitor Treatment for Gout and Type 2 Diabetes

**DOI:** 10.1001/jamanetworkopen.2023.30885

**Published:** 2023-08-25

**Authors:** Jie Wei, Hyon K. Choi, Nicola Dalbeth, Xiaoxiao Li, Changjun Li, Chao Zeng, Guanghua Lei, Yuqing Zhang

**Affiliations:** 1 Health Management Center, Xiangya Hospital, Central South University, Changsha, China; 2 Department of Orthopaedics, Xiangya Hospital, Central South University, Changsha, China; 3 Department of Epidemiology and Health Statistics, Xiangya School of Public Health, Central South University, Changsha, China; 4 Hunan Key Laboratory of Joint Degeneration and Injury, Changsha, China; 5 Key Laboratory of Aging-Related Bone and Joint Diseases Prevention and Treatment, Ministry of Education, Xiangya Hospital, Central South University, Changsha, China; 6 Division of Rheumatology, Allergy, and Immunology, Department of Medicine, Massachusetts General Hospital, Harvard Medical School, Boston; 7 The Mongan Institute, Massachusetts General Hospital, Harvard Medical School, Boston; 8 Department of Medicine, University of Auckland, Auckland, New Zealand; 9 Department of Endocrinology, Xiangya Hospital, Central South University, Changsha, China; 10 National Clinical Research Center for Geriatric Disorders, Xiangya Hospital, Central South University, Changsha, China

## Abstract

**Importance:**

Recurrent flares are the hallmark of clinical manifestation of gout. Sodium-glucose cotransporter-2 inhibitors (SGLT2i) have been associated with a lower risk of incident gout; however, their association with recurrent flares is unknown.

**Objective:**

To examine the association of SGLT2i vs active comparators (ie, glucagonlike peptide-1 receptor agonists [GLP-1 RA] or dipeptidyl peptidase-4 inhibitors [DPP-4i]) with the risk of recurrent gout flares and all-cause mortality among patients with gout and type 2 diabetes.

**Design, Setting, and Participants:**

This population-based retrospective cohort study was performed from January 1, 2013, to March 31, 2022, using a UK primary care database. Participants included patients with gout and type 2 diabetes with visits to their general practitioners.

**Exposures:**

Initiation of treatment with SGLT2i or active comparators.

**Main Outcomes and Measures:**

The primary outcome was the number of recurrent gout flares ascertained using recorded codes and prescription records. Secondary outcomes were the first recurrent gout flare and all-cause mortality. The association of SGLT2i compared with active comparators for the risk of recurrent flares, the first recurrent flare, and all-cause mortality was assessed using Poisson regression or the Cox proportional hazards model with propensity score overlap weighting.

**Results:**

Of a total of 5931 patients included in the analysis (mean [SD] age, 66.0 [11.6] years; 4604 [77.6%] men), 1548 initiated SGLT2i treatment and 4383 initiated treatment with active comparators during the study period. The relative rate of the recurrent flares with SGLT2i vs active comparators was 0.79 (95% CI, 0.65-0.97). Similar results were observed in the association of SGLT2i with the rate of recurrent flares when compared with DPP-4i or GLP-1 RA. For the first recurrent flare for SGLT2i vs active comparators, rate difference was −8.8 (95% CI, −17.2 to −0.4) per 1000 person-years and the hazard ratio was 0.81 (95% CI, 0.65-0.98). All-cause mortality per 1000 person-years was 18.8 for SGLT2i and 24.9 for active comparators, with rate difference of −6.1 (95% CI, −10.6 to −1.6) per 1000 person-years and hazard ratio of 0.71 (95% CI, 0.52-0.97).

**Conclusions and Relevance:**

The findings of this cohort study suggest that SGLT2i were associated with a lower risk of recurrent gout flares and mortality than their active comparators in patients with gout and type 2 diabetes. These findings further suggest that SGLT2i could help reduce the burden of recurrent gout flares and could also narrow the mortality gap between patients with gout and the general population.

## Key Points

**Question:**

What is the association between using sodium-glucose contransporter-2 inhibitors (SGLT2i) and the risk of recurrent gout flares among adults with gout and type 2 diabetes?

**Findings:**

In this cohort study of 5931 patients with gout and type 2 diabetes, initiation of SGLT2i treatment was associated with 19% fewer recurrent gout flares and 29% lower mortality than initiation of active comparator treatments.

**Meaning:**

These findings suggest that SGLT2i may reduce the burden of recurrent gout flares and narrow the mortality gap between patients with gout and the general population.

## Teaser

This cohort study compares the risk of recurrent gout flares and all-cause mortality between patients with gout and type 2 diabetes who initiate treatment with sodium-glucose cotransporter-2 inhibitors and those who initiate treatment with glucagonlike peptide-1 receptor agonists or dipeptidyl peptidase-4 inhibitors.

## Introduction

Gout is the most common inflammatory arthritis. The prevalence and incidence of gout have risen worldwide for decades.^1^ Although several efficacious pharmacologic regimens are available, many patients with gout continue to experience recurrent gout flares,^2^ which cause excruciating pain and morbidity^3,4^ and significantly affect activities of daily living.^5^ Gout also carries substantial comorbidity burden from cardiovascular-metabolic conditions.^6,7^ Furthermore, a number of studies have reported that gout increases the risk of death from kidney and cardiovascular disease,^8^ and the secular trend of the mortality gap between patients with gout and the general population remains unimproved.^9^

Current rheumatology guidelines recommend that patients with gout receive long-term therapy for lowering of urate levels (ULT) to achieve crystal dissolution, ultimately preventing recurrent gout flares.^10,11^ However, previous studies have shown that management of gout is still suboptimal, as many patients either do not receive ULT or do not receive the proper dose of ULT.^12,13^ In addition, adherence to ULT is low,^14^ resulting in recurrent gout flares.^15,16,17,18^

Sodium-glucose cotransporter-2 inhibitors (SGLT2i), which treat type 2 diabetes,^19^ reduce the major adverse cardiovascular events and all-cause mortality in individuals with or without diabetes.^20,21^ Studies also found that SGLT2i lower serum urate levels and reduce the risk of incident gout.^22,23,24,25,26,27,28,29^ However, to date, no study has specifically examined the association of SGLT2i with the risk of recurrent gout flares and all-cause mortality in patients with gout. Using an electronic medical records database from the UK, we conducted a population-based cohort study to compare the risk of recurrent gout flares and all-cause mortality between patients initiating SGLT2i treatment and those initiating 2 other antidiabetic medications (ie, glucagonlike peptide-1 receptor agonists [GLP-1 RA] or dipeptidyl peptidase-4 inhibitors [DPP-4i]) in patients with gout and type 2 diabetes.

## Methods

### Data Source

In this cohort study, we used data from the IQVIA Medical Research Database (IMRD), which incorporated data from The Health Improvement Network, a Cegedim database from general practitioners (GPs) in the UK. The IMRD contains health information on approximately 19 million patients from 839 general practices in the UK. The computerized information includes sociodemographic characteristics, anthropometric characteristics, lifestyle factors, details from visits to GPs, diagnoses from specialist referrals and hospital admissions, as well as results of laboratory tests. The Read classification system is used to code specific diagnoses, whereas a dictionary based on the Multilex classification system is used to code drugs. This study received approval from the medical ethical committee of Xiangya Hospital, with a waiver of informed consent owing to the use of deidentified data, and was approved by the IMRD Scientific Review Committee. The study followed the recommendations of the Strengthening the Reporting of Observational Studies in Epidemiology (STROBE) reporting guideline.

### Study Design and Cohort Definition

We compared the risk of recurrent gout flares between patients initiating SGLT2i treatment and those initiating GLP-1 RA or DPP-4i treatment.^30^ We selected DPP-4i and GLP-1 RA as the active comparators because they are novel second-line antidiabetic agents that are similar to SGLT2i and are known to have a neutral effect on serum urate levels in patients with type 2 diabetes.^31,32^ We included participants aged 18 to 89 years who had gout and type 2 diabetes from January 1, 2013, to December 31, 2021, and had at least 1 year of continuous enrollment with a general practice prior to entering the study. The diagnosis of gout and type 2 diabetes were based on at least 1 Read code for gout or type 2 diabetes.^33,34^ We identified initiators of SGLT2i or active comparators based on whether the first record of the prescription in the IMRD and the prescription date were later than the diagnosis of both gout and type 2 diabetes. The date of the first prescription of either SGLT2i or active comparators was assigned as the index date. We excluded individuals who had a cancer diagnosis or had been prescribed colchicine or the comparators during the year before the index date.

### Assessment of Outcomes

The primary outcome was the total number of recurrent gout flares (hereinafter referred to as recurrent flares) during the follow-up period. A gout flare was defined as a recorded Read code of gout plus a recorded prescription of colchicine; a recorded Read code of gout together with at least 1 of intra-articular corticosteroids, nonsteroidal anti-inflammatory drugs, or corticosteroid or adrenocorticotropic hormone within 1 week; or having Read codes specific for gout flare.^35,36^ The secondary outcomes consisted of the first recurrent gout flare (hereinafter referred to as the first recurrent flare) and all-cause mortality during the follow-up period.

### Assessment of Covariates

Covariates included age, sex, socioeconomic deprivation index score (measured using the Townsend Deprivation Index, which was grouped into quintiles from 1 [least deprived] to 5 [most deprived]), region, body mass index (BMI; calculated as weight in kilograms divided by height in meters squared), alcohol use, smoking, gout duration (year from the first gout diagnosis to the index date), type 2 diabetes duration (year from the first type 2 diabetes diagnosis to the index date), Charlson Comorbidity Index, comorbidities at any time since enrolment to the index date, medication use, and use of health care services (number of hospitalizations, visits to general practice, and referral to specialists) during the 1 year before the index date. The details of comorbidities and medication use are listed in Table 1.

**Table 1.** Baseline Characteristics by Initiation of Treatment With SGLT2i or Active Comparators Among Patients With Gout and Type 2 Diabetes

Variable	Patient cohort^a^					
	Before overlap weighting			After overlap weighting		
	SGLT2i (n = 1548)	Active comparators (n = 4383)	Standard difference	SGLT2i (n = 1548)	Active comparators (n = 4383)	Standard difference
Demographic						
Age, mean (SD), y	61.8 (10.6)	67.5 (11.6)	0.518	62.5 (10.0)	62.5 (11.7)	<0.001
Sex						
Women	248 (16.0)	1078 (24.6)	0.215	279 (18.0)	788 (18.0)	<0.001
Men	1300 (84.0)	3305 (75.4)	0.215	1269 (82.0)	3595 (82.0)	<0.001
Socioeconomic deprivation index score, mean (SD)^b^	3.1 (1.3)	3.0 (1.3)	0.076	3.0 (1.4)	3.0 (1.4)	<0.001
BMI, mean (SD)	34.8 (6.6)	33.1 (6.7)	0.249	34.6 (6.4)	34.6 (7.2)	<0.001
Serum urate level, mean (SD), μmol/L	388.9 (103.2)	400.0 (110.8)	0.104	391.1 (100.3)	391.1 (104.8)	<0.001
Gout duration, mean (SD), y	11.9 (8.1)	11.3 (8.6)	0.070	11.1 (7.7)	11.1 (7.7)	<0.001
Type 2 diabetes duration, mean (SD), y	8.2 (6.4)	9.0 (6.6)	0.118	7.9 (6.0)	7.9 (6.0)	<0.001
Region						
England	487 (31.5)	2218 (50.6)	0.434	635 (41.0)	1795 (41.0)	<0.001
Northern Ireland	187 (12.1)	359 (8.2)		175 (11.3)	494 (11.3)	
Scotland	442 (28.6)	688 (15.7)		285 (18.5)	811 (18.5)	
Wales	432 (27.9)	1118 (25.5)		453 (29.3)	1283 (29.3)	
Lifestyle factors						
Alcohol consumption						
None	271 (17.5)	956 (21.9)	0.131	268 (17.3)	758 (17.3)	<0.001
Past	105 (6.8)	250 (5.8)		90 (5.8)	254 (5.8)	
Current	1172 (75.7)	3177 (72.5)		1190 (76.9)	3371 (76.9)	
Smoking						
None	802 (51.8)	2135 (48.7)	0.066	788 (50.9)	2230 (50.9)	<0.001
Past	596 (38.5)	1828 (41.7)		636 (41.1)	1802 (41.1)	
Current	150 (9.7)	420 (9.6)		124 (8.0)	351 (8.0)	
Charlson Comorbidity Index, mean (SD)^c^	1.3 (1.2)	1.6 (1.5)	0.197	1.3 (1.2)	1.3 (1.2)	<0.001
Comorbidity						
Hypertension	1081 (69.8)	487 (11.1)	0.170	1101 (71.1)	3117 (71.1)	<0.001
Myocardial infarction	189 (12.2)	478 (10.9)	0.042	141 (9.1)	399 (9.1)	<0.001
Stroke	62 (4.0)	324 (7.4)	0.146	57 (3.7)	162 (3.7)	<0.001
Transient ischemic attack	57 (3.7)	250 (5.7)	0.096	63 (4.1)	180 (4.1)	<0.001
Congestive heart failure	186 (12.0)	500 (11.4)	0.018	122 (7.9)	346 (7.9)	<0.001
Ischemic heart disease	367 (23.7)	1100 (25.1)	0.033	324 (20.9)	916 (20.9)	<0.001
Osteoarthritis	412 (26.6)	1447 (33.0)	0.141	440 (28.4)	1245 (28.4)	<0.001
Varicose veins	50 (3.2)	302 (6.9)	0.071	94 (6.1)	267 (6.1)	<0.001
Venous thromboembolism	53 (3.4)	250 (5.7)	0.109	65 (4.2)	184 (4.2)	<0.001
Chronic kidney disease	166 (10.7)	1482 (33.8)	0.579	173 (11.2)	491 (11.2)	<0.001
Medication during the past year						
Antihypertensives	1246 (80.5)	3770 (86.0)	0.148	1248 (80.6)	3534 (80.6)	<0.001
Other antidiabetic medication	1485 (95.9)	4349 (99.2)	0.213	1515 (97.9)	4292 (97.9)	<0.001
Corticosteroid	362 (23.4)	1070 (24.4)	0.025	354 (22.9)	1004 (22.9)	<0.001
Aspirin	423 (27.3)	1495 (34.1)	0.147	401 (25.9)	1135 (25.9)	<0.001
PPIs	740 (47.8)	2056 (46.9)	0.017	738 (47.7)	2091 (47.7)	<0.001
Nitrates	142 (9.2)	412 (9.4)	0.004	107 (6.9)	302 (6.9)	<0.001
ULT	669 (43.2)	1946 (44.4)	0.025	734 (47.4)	2078 (47.4)	<0.001
Opioids	245 (15.8)	710 (16.2)	0.010	240 (15.5)	680 (15.5)	<0.001
NSAIDs	601 (38.8)	1850 (42.2)	0.071	636 (41.1)	1802 (41.1)	<0.001
Thiazide diuretics	194 (12.5)	706 (16.1)	0.103	217 (14.0)	614 (14.0)	<0.001
Loop diuretics	245 (15.8)	1074 (24.5)	0.218	234 (15.1)	662 (15.1)	<0.001
Health care service used in past year, mean (SD) No.						
Hospitalizations	0.5 (1.4)	0.7 (1.6)	0.124	0.5 (1.2)	0.5 (1.2)	<0.001
General practice visits	6.4 (6.1)	7.7 (7.2)	0.181	7.0 (6.1)	7.0 (6.4)	<0.001
Specialist referrals	0.6 (1.2)	0.8 (1.3)	0.119	0.7 (1.3)	0.7 (1.1)	<0.001

Abbreviations: BMI, body mass index (calculated as weight in kilograms divided by height in meters squared); NSAIDs, nonsteroidal anti-inflammatory drugs; PPIs, proton pump inhibitors; SGLT2i, sodium-glucose cotransporter-2 inhibitors; ULT, therapy to lower urate levels.

^a^ Unless otherwise indicated, data are expressed as No. (%) of patients. Active comparators consist of glucagonlike peptide-1 receptor agonists and dipeptidyl peptidase-4 inhibitors.

^b^ Measured by the Townsend Deprivation Index, which was grouped into quintiles from 1 (least deprived) to 5 (most deprived).

^c^ Measured by a weighted score of 17 comorbidities, which ranged from 0 (none) to 37. Higher scores indicate higher predicted mortality rate.

### Statistical Analysis

Participants were allocated into one of the nine 1-year blocks (ie, 2013-2021) based on the date of initiation of either SGLT2i or active comparator treatment. Within each 1-year time block, we assembled a cohort of SGLT2i initiators and a cohort of initiators of active comparators. In each 1-year time block, we calculated propensity scores for SGLT2i initiation conditional on the aforementioned baseline characteristics. We applied overlap weighting of the propensity scores to balance baseline characteristics between the comparison groups.^30^ The overlap weight is defined as 1 minus the propensity score for a treated unit and a propensity score for a comparison unit. Thus, patients with a propensity score of 0.5 make the largest contribution to the effect estimate, and patients with a propensity score close to 0 or 1 make the smallest contribution, therefore smoothly reducing the influence of patients at the tails of the propensity score distribution without making any exclusions and eliminating the potential bias that can arise from the multiplication of scores of the few patients with extremely high or low propensity scores. We assessed the distribution of baseline characteristics between 2 comparison cohorts before and after overlap weighting using the absolute standardized differences. Participants were followed up from the day of initiating medication under the study to the first of the following events to occur: disenrolled from a GP, age of 90 years, death, or the end of the study (ie, March 31, 2022). We calculated the weighted incidence rate for the recurrent flares and estimated the weighted absolute rate difference (RD) between 2 comparison cohorts. We calculated the relative rate (RR) and its 95% CI using a Poisson regression model. Missing values of BMI, smoking status, alcohol consumption, and socioeconomic deprivation index score were imputed using a sequential regression method. To minimize random error, we imputed 5 data sets using Rubin rules.^37^

We performed several sensitivity analyses to assess the robustness of the study findings. First, we compared the rate of recurrent flares between patients who initiated SGLT2i treatment and each of the active comparators (ie, DPP-4i and GLP-1 RA). Second, we conducted an as-treated analysis to account for nonadherence to medications under investigation. Specifically, we censored the follow-up at the time when participants either changed (eg, switched from SGLT2i to active comparators or vice versa) or discontinued (ie, no prescription refill for the respective class of medication with a period of >60 days) their initiated medication. Third, we conducted a complete data analysis by excluding participants who had missing data values of covariates to assess whether the missing data affect the study findings. Fourth, we performed 3 subgroup analyses stratifying the study sample according to sex, the ULT use during the 1 year before the index date (yes and no), and the baseline serum urate level (<360 μmol/L and ≥360 μmol/L). Fifth, we assessed the risk of recurrent gout flares during 1 year after initiating allopurinol in patients with gout. Sixth, we performed an analysis by including the level of serum urate at baseline in the propensity score estimation.

To examine whether the risk of the recurrent flares increased during the early period after initiating either SGLT2i or its active comparators, we estimated the monthly risk of the recurrent flares over the first 12 months. For the first recurrent flare analysis, the follow-up ended at the first of the following events: the occurrence of the first recurrent flare, disenrolled from a GP, age of 90 years, death, or the end of the study. We calculated the weighted incidence rate for the first recurrent flare and estimated the RD between 2 comparison cohorts. We performed a Cox proportional hazards model analysis to obtain a hazard ratio (HR) and its 95% CI of the first recurrent flare. We adopted the subdistribution hazard function to account for the competing event of death.^38^ Finally, we examined the association of SGLT2i vs active comparators with the risk of all-cause mortality using the Cox proportional hazards model. We also compared the risk of all-cause mortality between SGLT2i initiators and each of the active comparators separately.

All *P* values were 2-sided, and *P* < .05 was considered statistically significant for all tests. All statistical analyses were performed with SAS software, version 9.4 (SAS Institute Inc).

## Results

Among the 5931 patients included in the analysis (mean [SD] age, 66.0 [11.6] years; 1327 [22.4%] women and 4604 [77.6%] men), we identified 1548 patients (26.1%) who initiated SGLT2i treatment (mean [SD] age, 61.8 [10.6] years; 1300 [84.0%] men and 248 [16.0%] women; including 684 [44.2%] dapagliflozin, 613 [39.6%] empagliflozin, and 251 [16.2%] canagliflozin initiators) and 4383 (73.9%) who initiated of treatment with active comparators (mean [SD] age, 67.5 [11.6] years; 3305 [75.4%] men and 1078 [24.6%] women; 4059 [92.6%] DPP-4i and 324 [7.4%] GLP-1 RA initiators). Before propensity score overlap weighting, compared with those initiating treatment with active comparators, those initiating SGLT2i treatment were younger and more likely to be men; had higher BMI and lower levels of serum urate; had a lower prevalence of hypertension, stroke, osteoarthritis, venous thromboembolism, chronic kidney disease, and prescriptions of antihypertensive medicine, other antidiabetic medicine, aspirin, and diuretics; and less use of health care services. After propensity score overlap weighting, the distribution of the characteristics of the 2 comparison cohorts was well balanced (all standardized differences <0.001) (Table 1). The flowchart depicting the selection process of included patients is shown in eFigure 1 in Supplement 1. To compare the risk of recurrent flares between SGLT2i and each of the active comparators, we reassembled the cohorts by excluding only the specific comparators during 1 year before the index date. The flowcharts depicting the selection process of included patients are shown in eFigures 2 and 3 in Supplement 1. For the comparison between SGLT2i and DPP-4i, we identified 1829 initiators of SGLT2i with 4113 initiators of DPP-4i. Additionally, for the comparison between SGLT2i and GLP-1 RA, we identified 2551 initiators of SGLT2i with 787 initiators of GLP-1 RA. The baseline characteristics for participants initiating SGLT2i vs each of the active comparators are shown in eTables 1 and 2 in Supplement 1.

**Figure 1.**
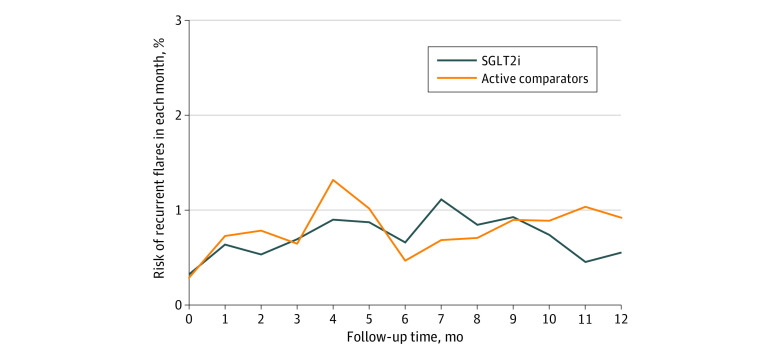
Risk of Recurrent Gout Flares in Each Month Over 12-Month Period After Initiation of Treatment With Sodium-Glucose Cotransporter-2 Inhibitors (SGLT2i) or Active Comparators Active comparators consist of glucagonlike peptide-1 receptor agonists or dipeptidyl peptidase-4 inhibitors.

As shown in Figure 1, there was no apparent transient increase of the risk of number of recurrent flares in the first year after initiation of either SGLT2i or active comparator treatments. For gout flares identified by a recorded Read code of gout along with treatments within 1 week, approximately 86% had a documented code for gout on the same date as a prescription of nonsteroidal anti-inflammatory drugs or corticosteroids, and the remaining patients had a recorded code for gout within 7 days before the prescription. The weighted incidence rate was lower in the SGLT2i cohort than in the comparison cohort (78.6 vs 99.0 per 1000 person-years). The weighted RD of the recurrent flares in the SGLT2i cohort vs the comparison cohort was −20.4 (95% CI, −39.6 to −1.2) per 1000 person-years; the RR, 0.79 (95% CI, 0.65-0.97) (Table 2). The weighted RR generated from the as-treated analysis was 0.80 (95% CI, 0.63-1.03); from the complete data analysis, 0.79 (95% CI, 0.64-0.98); and adjusting for baseline serum urate analysis, 0.80 (95% CI, 0.65-0.98). The associations of SGLT2i initiation with the lower risk of recurrent gout flares were consistent across different subgroups (eTable 3 in Supplement 1). We also observed fewer recurrent flares, albeit statistically insignificant, in the SGLT2i initiators than DPP-4i initiators (RR, 0.85 [95% CI, 0.71-1.02]). A similar result was observed when SGLT2i was compared with GLP-1 RA (RR, 0.65 [95% CI, 0.51-0.83]) (Table 2). Furthermore, the risk of gout flares increased during the first 3 months after allopurinol initiation and then leveled off (eFigure 4 in Supplement 1).

**Table 2.** Risk of Recurrent Gout Flares by Initiation of Either SGLT2i or Active Comparators Among Patients With Gout and Type 2 Diabetes

Variable	Treatment group	
	SGLT2i	Active comparator^a^
**All SGLT2i cohort (n = 1548) vs all active comparator cohort (n = 4383)**		
Event, No. of recurrent flares	318	1714
1	79	338
2	21	109
3	13	51
4	9	36
≥5	13	94
Weighted mean follow-up, y	2.76	2.70
Weighted rate of event per 1000 person-years	78.6	99.0
Weighted RD (95% CI) per 1000 person-years	−20.4 (−39.6 to −1.2)	0 [Reference]
Age-, sex-, and entry year–adjusted RR (95% CI)	0.76 (0.67 to 0.86)	1 [Reference]
Weighted RR (95% CI)	0.79 (0.65 to 0.97)	1 [Reference]
**SGLT2i cohort (n = 1829) vs DPP-4i cohort (n = 4113)**		
Total No. of recurrent flares	428	1599
Weighted mean follow-up, y	2.95	2.86
Weighted rate of event per 1000 person-years	85.7	93.7
Weighted RD (95% CI) per 1000 person-years	−8.1 (−17.0 to 0.8)	0 [Reference]
Age-, sex-, and entry year–adjusted RR (95% CI)	0.85 (0.75 to 0.95)	1 [Reference]
Weighted RR (95% CI)	0.85 (0.71 to 1.02)	1 [Reference]
**SGLT2i cohort (n = 2551) vs GLP-1 RA cohort (n = 787)**		
Total No. of recurrent flares	539	375
Weighted mean follow-up (years)	2.94	2.91
Weighted rate of event, per 1000 person-years	88.9	143.8
Weighted RD (95% CI), per 1000 person-years	−54.9 (−84.4 to −25.3)	0 [Reference]
Age-, sex-, and entry year–adjusted RR (95% CI)	0.54 (0.47 to 0.62)	1 [Reference]
Weighted RR (95% CI)	0.65 (0.51 to 0.83)	1 [Reference]

Abbreviations: DPP-4i, dipeptidyl peptidase-4 inhibitors; GLP-1 RA, glucagonlike peptide-1 receptor agonist; RD, rate differences; RR, relative rate; SGLT2i, sodium-glucose cotransporter-2 inhibitors.

^a^ Active comparators consist of DPP-4i and GLP-1 RA.

**Figure 2.**
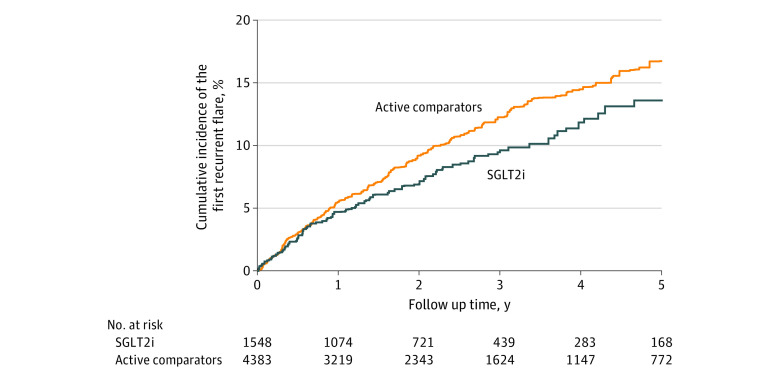
Cumulative Incidence of the First Recurrent Gout Flare After Initiation of Treatment With Sodium-Glucose Cotransporter-2 Inhibitors (SGLT2i) or Active Comparators Active comparators consist of glucagonlike peptide-1 receptor agonists or dipeptidyl peptidase-4 inhibitors.

As shown in Figure 2 and Table 3, the weighted incidence rate of the first recurrent flare was 19% lower in the SGLT2i cohort than in the comparison cohort (32.4 vs 41.2 per 1000 person-years). The weighted RD of the first recurrent flare in the SGLT2i cohort vs the comparison cohort was −8.8 (95% CI, −17.2 to −0.4) per 1000 person-years, and the weighted HR of the first recurrent flare was 0.81 (95% CI, 0.65-0.98).

**Table 3.** Risk of the First Recurrent Gout Flares and All-Cause Mortality by Initiation of Either SGLT2i or Active Comparators Among Patients With Gout and Type 2 Diabetes

Outcome	Treatment cohort	
	SGLT2i (n = 1548)	Active comparators (n = 4383)^a^
**First recurrent gout flares**		
Event, No.	135	628
Weighted mean follow-up, y	2.57	2.46
Weighted rate of event per 1000 person-years	32.4	41.2
Weighted RD (95% CI) per 1000 person-years	−8.8 (−17.2 to −0.4)	0 [Reference]
Age-, sex-, and entry year–adjusted HR (95% CI)	0.78 (0.64 to 0.95)	1 [Reference]
Weighted HR (95% CI)	0.81 (0.65 to 0.98)	1 [Reference]
**All-cause mortality**		
Event, No.	63	607
Weighted mean follow-up, y	2.76	2.70
Weighted rate of event per 1000 person-years	18.8	24.9
Weighted RD (95% CI) per 1000 person-years	−6.1 (−10.6 to −1.6)	0 [Reference]
Age-, sex-, and entry year–adjusted HR (95% CI)	0.67 (0.51 to 0.89)	1 [Reference]
Weighted HR (95% CI)	0.71 (0.52 to 0.97)	1 [Reference]

Abbreviations: HR, hazard ratio; RD, rate difference; SGLT2i, sodium-glucose cotransporter-2 inhibitors.

^a^ Active comparators consist of dipeptidyl peptidase-4 inhibitors or glucagonlike peptide-1 receptor agonist.

As shown in eFigure 5 in Supplement 1 and Table 3, all-cause mortality was 29% lower in the SGLT2i cohort than that in the comparison cohort (18.8 vs 24.9 per 1000 person-years; RD, −6.1 [95% CI, −10.6 to −1.6] per 1000 person-years; HR, 0.71 [95% CI, 0.52-0.97]). Compared with the active comparators, the HRs of all-cause mortality for SGLT2i were 0.68 (95% CI, 0.39-1.19) for 1-year follow-up, 0.85 (95% CI, 0.56-1.28) for 2-year follow-up, 0.78 (95% CI, 0.55-1.33) for 3-year follow-up, 0.73 (95% CI, 0.52-1.02) for 4-year follow-up, and 0.71 (95% CI, 0.51-0.97) for 5-year follow-up. The lower risk of all-cause mortality among the initiators of SGLT21 was mainly seen when compared with the initiators of DPP-4i (HR, 0.63 [95% CI, 0.48-0.84]) but not GLP-1 RA (HR, 1.12 [95% CI, 0.89-1.52]).

## Discussion

Using the UK population-based database, we found that initiating SGLT2i treatment was associated with a lower risk of recurrent gout flares among the patients with gout and type 2 diabetes. Unlike other ULT medications, we did not observe an apparent transient increase in the risk of gout flares shortly after initiating SGLT2i therapy. In addition, SGLT2i initiators had lower all-cause mortality than DPP-4i initiators.

Previous post hoc analyses of randomized clinical trials^23,24,25,27^ showed that SGLT2i reduced serum urate level, incident hyperuricemia, and incident gout in patients with type 2 diabetes or chronic heart failure, compared with placebo. Several observational studies^22,26,28,29^ also reported that initiation of SGLT2i treatment was associated with a lower risk of incident gout than initiation of DPP-4i or GLP-1 RA treatment in patients with type 2 diabetes. However, to our knowledge, no study has previously assessed whether SGLT2i reduces the risk of recurrent gout flares. Our study fills this knowledge gap and demonstrates that initiating SGLT2i treatment was associated with a lower rate of recurrent flares than initiating DPP-4i or GLP-1 RA treatment in patients with gout and type 2 diabetes.

Several biological mechanisms may explain our findings. First, SGLT2i could reduce serum urate concentration by increasing kidney urate elimination.^39^ This effect is attributed to the glucose in the urine competing with soluble urate for glucose transporter 9–mediated reabsorption in the proximal tubule due to the reduced transport function of SGLT2.^40^ Additionally, SGLT2i enhance sirtuin-1, an enzyme that inhibits xanthine oxidase and decreases serum urate levels.^41^ Second, SGLT2i may suppress pyrin domain–containing 3 inflammasome activation and attenuate interleukin 1β secretion^42^ and thus may lower the risk of gout flares. Finally, studies have shown that SGLT2i could improve kidney function and heart failure and reduce the use of loop or thiazides diuretics,^19,20,43^ which may indirectly lower the risk of recurrent gout flares.

### Strengths and Limitations

Several strengths of our study are worth noting. First, we implemented an active-comparator new-user design to assess the risk of recurrent flares associated with SGLT2i use. This design helps minimize the confounding by indication and prevalent user biases. Second, no ULT medications have shown a protective effect on all-cause mortality in patients with gout. Although we did not observe a significant reduction of all-cause mortality during the early years of follow-up after initiating SGLT2i treatment, our findings suggest that SGLT2i may be associated with a reduced risk of all-cause mortality. This finding is consistent with previous studies that have shown a similar association,^21^ enhancing the credibility of our findings regarding the risk of gout flares. Third, we used the sequential overlap-weighting method to control for confounding; and the age- and sex-adjusted effect estimates are similar to those generated from the overlap-weighted method, indicating that any residual confounding, if present, is unlikely to explain away the association of SGLT2i with either the risk of recurrent gout flares or all-cause mortality.

This study also has some limitations. First, the IMRD does not contain hospitalization data, and some patients may not seek care from GPs for recurrent gout flares. Additionally, the ascertainment of gout flares using a pragmatic approach may lead to a misclassification. As a result, the risk of recurrent gout flare might be underestimated. Nevertheless, it is worth noting that, for gout flares identified by a recorded Read code of gout along with treatments within 1 week, approximately 86% of patients in our study had a documented code for gout on the same date as a prescription of nonsteroidal anti-inflammatory drugs or corticosteroids, and the remaining patients had a recorded code for gout within 7 days before the prescription. Although the therapeutic use of colchicine has extended to other disorders (eg, cardiovascular disease), we would expect patients to take colchicine continuously. When colchicine is prescribed for a discrete episode, it is more often to patients who experienced gout flares.^10,44^ In addition, we demonstrated that the risk of gout flares increased during the first 3 months after initiating allopurinol and then leveled off, which is comparable to the findings of the previous randomized clinical trials.^45^ All this evidence suggests a reasonable level of ascertainment for gout flares. Furthermore, if gout flares were misclassified, such misclassification is likely nondifferential and biases the results toward the null. Second, we could not evaluate the association of SGLT2i with the risk of recurrent gout flares and mortality among the population who seek care outside the GP system included in IMRD; however, this limitation should not affect the internal validity of the current findings. Third, physician-ordered prescriptions may not reflect the actual medication taken by the patients. However, we found that the results from the intention-to-treat analysis did not differ materially from those of the as-treated analysis, suggesting that the potential bias from nonadherence may not be substantial.

## Conclusions

In this cohort study of patients with gout and type 2 diabetes, initiating SGLT2i treatment was associated with a reduced risk of recurrent gout flares compared with initiating DPP-4i or GLP-1 RA treatment. Furthermore, SGLT2i initiators had a lower all-cause mortality than initiators of DPP-4i. These findings suggest that SGLT2i might hold potential in reducing the burden of recurrent gout flares and potentially narrowing the mortality gap between patients with gout and the general population.

## Notes

Supplement 1. **eTable 1.** Baseline Characteristics Among Patients With Gout and Type 2 Diabetes Initiating SGLT2i or DPP-4i **eTable 2.** Baseline Characteristics Among Patients With Gout and Type 2 Diabetes Initiating SGLT2i or GLP-1 RA **eTable 3.** Subgroup Analyses Stratified by Risk Factors for Recurrent Gout Flares **eFigure 1.** Selection Process of Included Patients With Gout and Type 2 Diabetes Initiating SGLT2i or Active Comparators **eFigure 2.** Selection Process of Included Patients With Gout and Type 2 Diabetes Initiating SGLT2i or DPP-4i **eFigure 3.** Selection Process of Included Patients With Gout and Type 2 Diabetes Initiating SGLT2i or GLP-1 RA **eFigure 4.** Risk of Recurrent Gout Flares in Each Month Over a 12-Month Period After Initiation of Allopurinol Among Patients With Gout and Type 2 Diabetes **eFigure 5.** Cumulative Incidence of All-Cause Mortality After Initiation of Either SGLT2i or Active Comparators

Supplement 2.
Data Sharing Statement
