# Supplementary material for: Gout Flares and Mortality After Sodium-Glucose Cotransporter-2 Inhibitor Treatment for Gout and Type 2 Diabetes
Source: JAMA Netw Open. 2023 Aug 25;6(8):e2330885. doi: 10.1001/jamanetworkopen.2023.30885 (PMC10457713; doi:10.1001/jamanetworkopen.2023.30885)
Supplement: Supplement 1. — eTable 1. Baseline Characteristics Among Patients With Gout and Type 2 Diabetes Initiating SGLT2i or DPP-4i eTable 2. Baseline Characteristics Among Patients With Gout and Type 2 Diabetes Initiating SGLT2i or GLP-1 RA eTable 3. Subgroup Analyses Stratified by Risk Factors for Recurrent Gout Flares eFigure 1. Selection Process of Included Patients With Gout and Type 2 Diabetes Initiating SGLT2i or Active Comparators eFigure 2. Selection Process of Included Patients With Gout and Type 2 Diabetes Initiating SGLT2i or DPP-4i eFigure 3. Selection Process of Included Patients With Gout and Type 2 Diabetes Initiating SGLT2i or GLP-1 RA eFigure 4. Risk of Recurrent Gout Flares in Each Month Over a 12-Month Period After Initiation of Allopurinol Among Patients With Gout and Type 2 Diabetes eFigure 5. Cumulative Incidence of All-Cause Mortality After Initiation of Either SGLT2i or Active Comparators [file jamanetwopen-e2330885-s001.pdf]

Supplementary Online Content

Wei J, Choi HK, Dalbeth N, et al. Gout flares and mortality after sodium-glucose cotransporter-2 inhibitor treatment for gout and type 2 diabetes. *JAMA Netw Open*. 2023;6(8):e2330885. doi:10.1001/jamanetworkopen.2023.30885

eTable 1. Baseline Characteristics Among Patients With Gout and Type 2 Diabetes Initiating SGLT2i or DPP-4i
eTable 2. Baseline Characteristics Among Patients With Gout and Type 2 Diabetes Initiating SGLT2i or GLP-1 RA
eTable 3. Subgroup Analyses Stratified by Risk Factors for Recurrent Gout Flares
eFigure 1. Selection Process of Included Patients With Gout and Type 2 Diabetes Initiating SGLT2i or Active Comparators
eFigure 2. Selection Process of Included Patients With Gout and Type 2 Diabetes Initiating SGLT2i or DPP-4i
eFigure 3. Selection Process of Included Patients With Gout and Type 2 Diabetes Initiating SGLT2i or GLP-1 RA
eFigure 4. Risk of Recurrent Gout Flares in Each Month Over a 12-Month Period After Initiation of Allopurinol Among Patients With Gout and Type 2 Diabetes
eFigure 5. Cumulative Incidence of All-Cause Mortality After Initiation of Either SGLT2i or Active Comparators

This supplementary material has been provided by the authors to give readers additional information about their work.

© 2023 Wei J et al. *JAMA Network Open*.

eTable 1. Baseline Characteristics Among Patients With Gout and Type 2 Diabetes Initiating SGLT2i or DPP-4i

Variable	Patient cohort[a]					
	Before overlap weighting			After overlap weighting		
	SGLT2i (n=1829)	DPP-4i (n=4113)	Standard difference	SGLT2i (n=1829)	DPP-4i (n=4113)	Standard difference
**Demographics**						
Age, mean (SD), y	61.7 (10.4)	68.2 (11.5)	0.59	63.0 (9.8)	63.0 (11.5)	<0.001
Socioeconomic deprivation index score, mean (SD)[b]	3.0 (1.3)	2.9 (1.3)	0.081	3.0 (1.4)	3.0 (1.4)	<0.001
Women (%)	313 (17.1)	1020 (24.8)	0.189	366 (20.0)	823 (20.0)	<0.001
Men (%)	1516 (82.9)	3093 (75.2)	0.189	1463 (80.0)	3290 (80.0)	<0.001
**BMI, mean (SD), kg/m^2**	35.3 (6.7)	32.7 (6.4)	0.389	34.6 (6.3)	34.6 (7.0)	<0.001
**Serum urate level, mean (SD), µmol/L**	394.9 (106.5)	406.2 (112.0)	0.104	395.7 (104.1)	395.7 (106.6)	<0.001
**Gout duration, mean (SD), y**	12.0 (8.3)	11.4 (8.7)	0.072	11.1 (7.9)	11.1 (8.1)	<0.001
**Type 2 duration, mean (SD), y**	9.0 (6.6)	9.1 (6.6)	0.019	8.4 (6.0)	8.4 (6.3)	<0.001
**Region**			0.426			<0.001
England	588 (32.2)	2106 (51.2)		774 (42.3)	1740 (42.3)	
Northern Ireland	207 (11.3)	345 (8.4)		194 (10.6)	436 (10.6)	
Scotland	504 (27.6)	617 (15.0)		303 (16.6)	683 (16.6)	
Wales	530 (29.0)	1045 (25.4)		558 (30.5)	1254 (30.5)	
**Lifestyle factors**						
Alcohol consumption			0.124			<0.001
None	318 (17.4)	917 (22.3)		317 (17.3)	713 (17.3)	
Past	125 (6.8)	239 (5.9)		103 (5.6)	232 (5.6)	
Current	1386 (75.8)	2957 (71.9)		1409 (77)	3168 (77)	
Smoking			0.057			<0.001
None	944 (51.5)	2006 (48.8)		933 (51)	2098 (51)	
Past	715 (39.2)	1717 (41.8)		748 (40.9)	1682 (40.9)	
Current	170 (9.3)	390 (9.4)		148 (8.1)	333 (8.1)	
**Charlson comorbidity index, mean (SD)[c]**	1.3 (1.2)	1.6 (1.5)	0.189	1.3 (1.2)	1.3 (1.2)	<0.001
**Comorbidity**						
Hypertension	1299 (71.0)	3200 (77.8)	0.157	1319 (72.1)	2965 (72.1)	<0.001
Myocardial infarction	214 (11.7)	465 (11.3)	0.014	166 (9.1)	374 (9.1)	<0.001
Stroke	77 (4.2)	313 (7.6)	0.147	71 (3.9)	160 (3.9)	<0.001

© 2023 Wei J et al. *JAMA Network Open.*

Transient ischemic attack	68 (3.7)	239 (5.8)	0.101	75 (4.1)	169 (4.1)	<0.001
Congestive heart failure	209 (11.4)	481 (11.7)	0.012	155 (8.5)	350 (8.5)	<0.001
Ischemic heart disease	433 (23.7)	1061 (25.8)	0.049	393 (21.5)	884 (21.5)	<0.001
Osteoarthritis	497 (27.2)	1370 (33.3)	0.133	541 (29.6)	1217 (29.6)	<0.001
Varicose veins	102 (5.6)	284 (6.9)	0.055	117 (6.4)	263 (6.4)	<0.001
Venous thromboembolism	79 (4.3)	243 (5.9)	0.073	82 (4.5)	185 (4.5)	<0.001
Chronic kidney disease	205 (11.2)	1431 (34.8)	0.587	238 (13)	535 (13)	<0.001
**Medication during the past year**						
Antihypertensive medicine	1507 (82.4)	3550 (86.3)	0.108	1505 (82.3)	3385 (82.3)	<0.001
Other antidiabetic medication	1767 (96.6)	4084 (99.3)	0.191	1800 (98.4)	4047 (98.4)	<0.001
Corticosteroid	422 (23.1)	1020 (24.8)	0.041	419 (22.9)	942 (22.9)	<0.001
Aspirin	523 (28.6)	1411 (34.3)	0.121	503 (27.5)	1131 (27.5)	<0.001
PPIs	898 (49.1)	1937 (47.1)	0.039	885 (48.4)	1991 (48.4)	<0.001
Nitrates	166 (9.1)	383 (9.3)	0.007	128 (7.0)	288 (7.0)	<0.001
Thiazide diuretics	245 (13.4)	658 (16.0)	0.075	269 (14.7)	605 (14.7)	<0.001
ULT	786 (43.0)	1810 (44.0)	0.02	860 (47)	1933 (47)	<0.001
Opioids	311 (17.0)	670 (16.3)	0.019	294 (16.1)	662 (16.1)	<0.001
NSAIDs	721 (39.4)	1756 (42.7)	0.067	757 (41.4)	1703 (41.4)	<0.001
Loop diuretics	300 (16.4)	1036 (25.2)	0.218	278 (15.2)	625 (15.2)	<0.001
**Health care service used in past year, mean (SD), No**						
Hospitalizations‡	0.5 (1.3)	0.7 (1.6)	0.142	0.5 (1.2)	0.5 (1.3)	<0.001
General practice visits‡	6.6 (6.3)	7.7 (7.2)	0.171	7.1 (6.2)	7.1 (6.6)	<0.001
Specialist referrals‡	0.6 (1.2)	0.8 (1.3)	0.114	0.8 (1.3)	0.8 (1.2)	<0.001

Abbreviations: BMI, body mass index (calculated as weight in kilograms divided by height in meters squared); SGLT2i, sodium glucose cotransporter-2 inhibitors; DPP-4i, dipeptidyl peptidase-4 inhibitors; NSAIDs, nonsteroidal anti-inflammatory drugs; PPIs, proton pump inhibitors; ULT, therapy to lower urate levels.

a Unless otherwise indicated, data are expressed as No. (%) of patients.

b Measured by the Townsend Deprivation Index, which was grouped into quintiles from 1 (least deprived) to 5 (most deprived).

c Charlson comorbidity index was measured by a weighted score of 17 comorbidities, which was ranged from 0 to 37. A score of zero means that no comorbidities were found, the higher the score, the higher the predicted mortality rate is.

© 2023 Wei J et al. *JAMA Network Open*.

eTable 2. Baseline Characteristics Among Patients With Gout and Type 2 Diabetes Initiating SGLT2i or GLP-1 RA

Variable	Patient cohort[a]					
	Before overlap weighting			After overlap weighting		
	SGLT2i (n=2551)	GLP-1 RA (n=787)	Standard difference	SGLT2i (n=2551)	GLP-1 RA (n=787)	Standard difference
**Demographics**						
Age, mean (SD), y	62.5 (10.5)	62.2 (10.0)	0.027	62.4 (10.9)	62.3 (10.2)	0.001
Socioeconomic deprivation index score, mean (SD)[b]	3.0 (1.3)	3.1 (1.3)	0.040	3.0 (1.3)	3.0 (1.3)	0.001
Women (%)	426 (16.7)	194 (24.7)	0.198	582 (22.8)	179 (22.8)	<0.001
Men (%)	2125 (83.3)	593 (75.3)	0.198	1969 (77.2)	608 (77.2)	<0.001
**BMI, mean (SD), kg/m^2**	34.3 (6.4)	37.6 (6.8)	0.495	36.4 (7.0)	36.4 (5.8)	0.001
**Serum urate level, mean (SD), µmol/L**	387.7 (104.1)	408.1 (113.7)	0.187	397.7 (101.8)	397.7 (106.6)	<0.001
**Gout duration, mean (SD), y**	12.2 (8.3)	11.2 (8.4)	0.119	10.8 (8.0)	10.8 (8.2)	<0.001
**Type 2 duration, mean (SD), y**	9.0 (6.3)	10.3 (7.2)	0.196	9.4 (6.5)	9.4 (6.0)	<0.001
**Region**			0.322			<0.001
England	866 (34)	371 (47.1)		1025 (40.2)	316 (40.2)	
Northern Ireland	291 (11.4)	44 (5.6)		185 (7.3)	58 (7.3)	
Scotland	640 (25.1)	147 (18.7)		464 (18.2)	142 (18.2)	
Wales	754 (29.6)	225 (28.6)		877 (34.4)	271 (34.4)	
**Lifestyle factors**						
Alcohol consumption			0.068			<0.001
None	441 (17.4)	150 (19.1)		522 (20.5)	161 (20.5)	
Past	161 (6.3)	39 (5.0)		114 (4.5)	35 (4.5)	
Current	1949 (76.3)	598 (75.9)		1915 (75.1)	591 (75.1)	
Smoking			0.067			<0.001
None	1306 (51.2)	385 (48.9)		1240 (48.6)	382 (48.6)	
Past	997 (39.1)	334 (42.3)		1093 (42.9)	338 (42.9)	
Current	247 (9.7)	68 (8.8)		218 (8.6)	67 (8.6)	
**Charlson comorbidity index, mean (SD)[c]**	1.3 (1.2)	1.5 (1.3)	0.143	1.3 (1.1)	1.3 (1.0)	<0.001
**Comorbidity**						
Hypertension	1837 (72.0)	600 (76.2)	0.098	1931 (75.7)	596 (75.7)	<0.001
Myocardial infarction	281 (11.0)	83 (10.5)	0.015	242 (10.5)	75 (10.5)	<0.001
Stroke	110 (4.3)	42 (5.3)	0.05	117 (4.6)	36 (4.6)	<0.001
Transient ischemic attack	94 (3.7)	38 (4.8)	0.057	99 (3.9)	31 (3.9)	<0.001
Congestive heart	255 (10.0)	86 (10.9)	0.03	217 (8.5)	67 (8.5)	<0.001

© 2023 Wei J et al. *JAMA Network Open*.

failure						
Ischemic heart disease	574 (22.5)	187 (23.8)	0.029	523 (20.5)	162 (20.6)	0.001
Osteoarthritis	689 (27)	250 (31.8)	0.105	796 (31.2)	246 (31.2)	0.001
Varicose veins	145 (5.7)	55 (7)	0.052	196 (7.7)	60 (7.6)	0.002
Venous thromboembolism	99 (3.9)	55 (7)	0.135	148 (5.8)	46 (5.8)	<0.001
Chronic kidney disease	296 (11.6)	222 (28.2)	0.425	536 (21)	166 (21)	0.001
**Medication during the past year**						
Antihypertensive medicine	2102 (82.4)	688 (87.4)	0.141	2217 (86.9)	684 (86.9)	<0.001
Other antidiabetic medication	2487 (97.5)	782 (99.4)	0.149	2531 (99.2)	781 (99.2)	<0.001
Corticosteroid	577 (22.6)	191 (24.3)	0.039	663 (26)	204 (25.9)	0.001
Aspirin	732 (28.7)	278 (35.3)	0.142	776 (30.4)	239 (30.4)	<0.001
PPIs	1222 (47.9)	397 (50.4)	0.051	1235 (48.4)	381 (48.4)	<0.001
Nitrates	224 (8.8)	64 (8.1)	0.025	166 (6.5)	52 (6.6)	0.002
Thiazide diuretics	355 (13.9)	130 (16.5)	0.074	408 (16)	126 (16)	<0.001
ULT	1082 (42.4)	372 (47.3)	0.098	1245 (48.8)	384 (48.8)	<0.001
Opioids	398 (15.6)	124 (19.6)	0.105	474 (18.6)	146 (18.6)	<0.001
NSAIDs	1000 (39.2)	326 (41.4)	0.046	1092 (42.8)	337 (42.8)	<0.001
Loop diuretics	378 (14.8)	202 (25.7)	0.273	490 (19.2)	164 (20.9)	0.043
**Health care service used in past year, mean (SD), No**						
Hospitalizations‡	0.5 (1.3)	0.5 (1.4)	0.034	0.4 (1.0)	0.4 (0.9)	<0.001
General practice visits‡	6.5 (6.2)	7.6 (7.1)	0.163	7.0 (6.2)	7.0 (5.9)	<0.001
Specialist referrals‡	0.6 (1.2)	0.9 (1.4)	0.178	0.8 (1.3)	0.8 (1.3)	<0.001

Abbreviations: BMI, body mass index (calculated as weight in kilograms divided by height in meters squared); SGLT2i, sodium glucose cotransporter-2 inhibitors; GLP-1 RA, glucagon-like peptide-1 receptor agonist; NSAIDs, nonsteroidal anti-inflammatory drugs; PPIs, proton pump inhibitors; ULT, therapy to lower urate levels.

a Unless otherwise indicated, data are expressed as No. (%) of patients.

b Measured by the Townsend Deprivation Index, which was grouped into quintiles from 1 (least deprived) to 5 (most deprived).

c Charlson comorbidity index was measured by a weighted score of 17 comorbidities, which was ranged from 0 to 37. A score of zero means that no comorbidities were found, the higher the score, the higher the predicted mortality rate is.

© 2023 Wei J et al. *JAMA Network Open*.

eTable 3. Subgroup Analyses Stratified by Risk Factors for Recurrent Gout Flares

Subgroup analyses	Number of participants	Number of recurrent flares	Weighted mean follow-up years	Weighted rate of event†	Weighted RD (95% CI) †	Weighted RR (95% CI)
**Stratified by sex**						
**Male**						
SGLT2i	1,300	288	2.77	83.4	0.0 (reference)	1.00 (reference)
Active comparators*	3,303	1,302	2.69	97.9	-14.5 (-28.8 to -0.2)	0.84 (0.67 to 0.99)
**Female**						
SGLT2i	248	30	2.13	107.9	0.0 (reference)	1.00 (reference)
Active comparators*	1,080	412	2.13	199.5	-91.6 (-174.5 to -8.7)	0.47 (0.24 to 0.92)
**Stratified by baseline ULT usage**						
**Yes**						
SGLT2i	669	107	2.64	55.6	0.0 (reference)	1.00 (reference)
Active comparators*	1,948	478	2.61	85.9	-30.3 (-57.7 to -2.9)	0.72 (0.56 to 0.93)
**No**						
SGLT2i	879	211	2.76	86.7	0.0 (reference)	1.00 (reference)
Active comparators*	2,435	1,236	2.71	103.8	-17.1 (-34.4 to 0.2)	0.91 (0.60 to 1.01)
**Stratified by baseline serum urate level#**						
**<360 μmol/L**						
SGLT2i	384	37	2.49	21.2	0.0 (reference)	1.00 (reference)
Active comparators*	1,041	221	2.47	65.2	-44.0 (-79.1 to -8.9)	0.44 (0.25 to 0.77)
**≥360 μmol/L**						
SGLT2i	615	171	2.50	99.5	0.0 (reference)	1.00 (reference)
Active comparators*	1,760	947	2.53	106.4	-6.9 (-14.5 to 0.7)	0.89 (0.78 to 1.05)

n, number; SGLT2i, sodium glucose cotransporter-2 inhibitors; ULT, urate-lowering therapy; RD, rate differences; RR, relative rate; 95% CI, 95% confidence interval.

© 2023 Wei J et al. *JAMA Network Open*.

*Active comparators consist of dipeptidyl peptidase 4 inhibitors or glucagon-like peptide-1 receptor agonist.

† Per 1000 person-years.

# Patients with missing baseline serum urate levels were excluded from the analysis.

© 2023 Wei J et al. *JAMA Network Open*.

eFigure 1. Selection Process of Included Patients With Gout and Type 2 Diabetes Initiating SGLT2i or Active Comparators

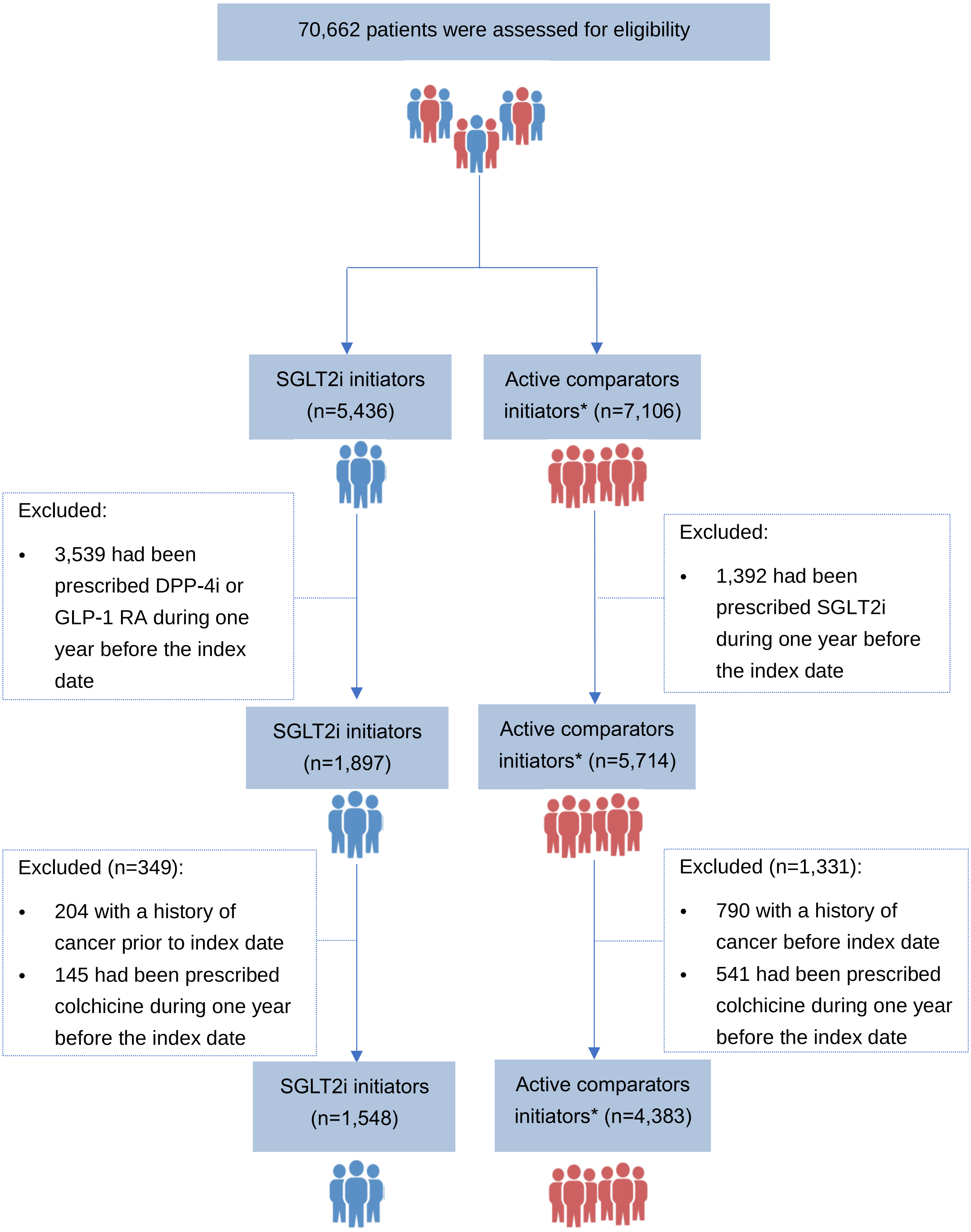

© 2023 Wei J et al. *JAMA Network Open*.

SGLT2i, sodium glucose cotransporter-2; DPP-4i, dipeptidyl peptidase 4 inhibitors; GLP-1 RA, glucagon-like peptide-1 receptor agonist. *Active comparators consist of dipeptidyl peptidase 4 inhibitor or glucagon-like peptide-1 receptor agonist.

© 2023 Wei J et al. *JAMA Network Open*.

eFigure 2. Selection Process of Included Patients With Gout and Type 2 Diabetes Initiating SGLT2i or DPP-4i

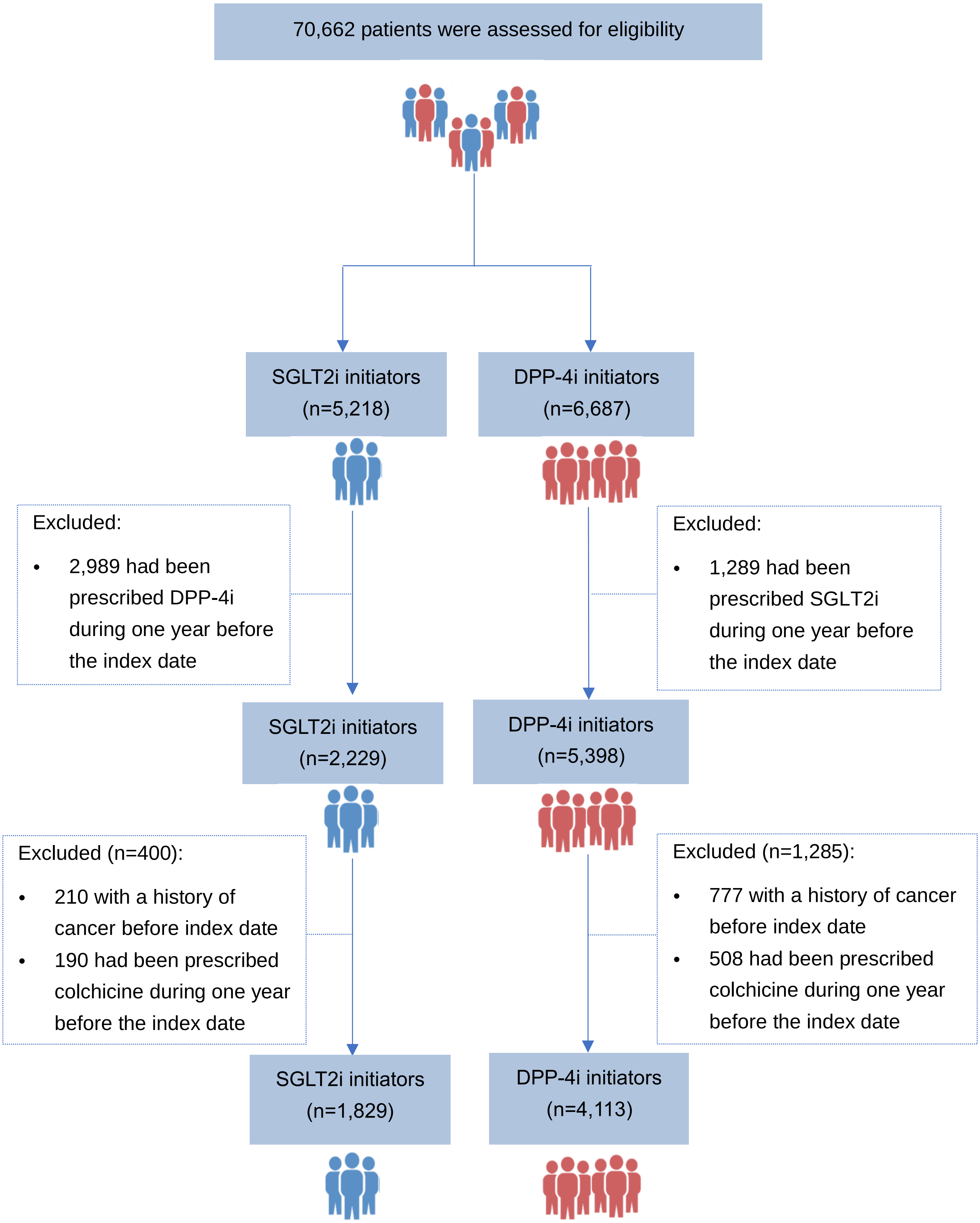

SGLT2i, sodium glucose cotransporter-2; DPP-4i, dipeptidyl peptidase-4 inhibitors.

© 2023 Wei J et al. *JAMA Network Open*.

eFigure 3. Selection Process of Included Patients With Gout and Type 2 Diabetes Initiating SGLT2i or GLP-1 RA

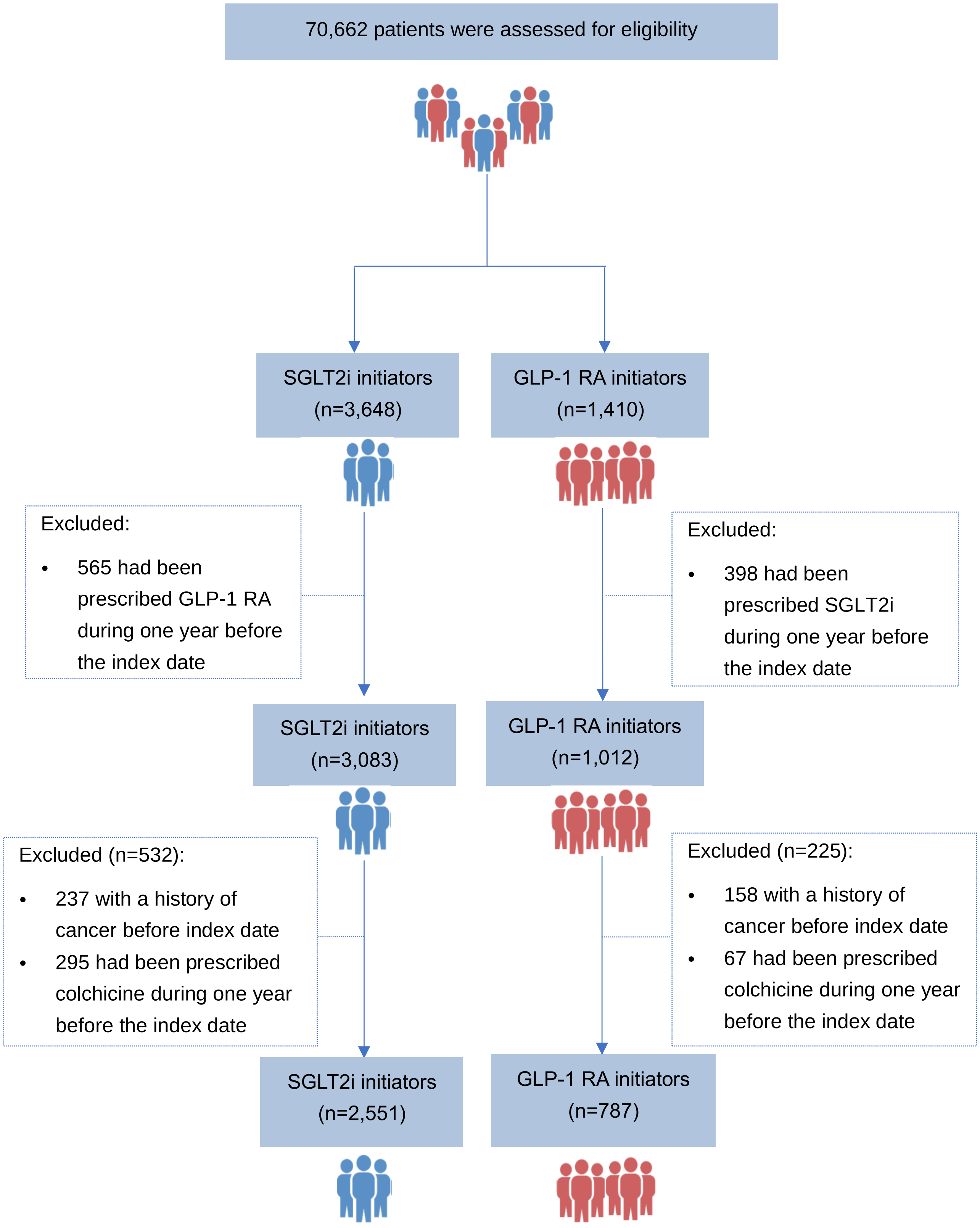

© 2023 Wei J et al. *JAMA Network Open*.

SGLT2i, sodium glucose cotransporter-2; GLP-1 RA, glucagon-like peptide-1 receptor agonist.

© 2023 Wei J et al. *JAMA Network Open*.

eFigure 4. Risk of Recurrent Gout Flares in Each Month Over a 12-Month Period After Initiation of Allopurinol Among Patients With Gout and Type 2 Diabetes

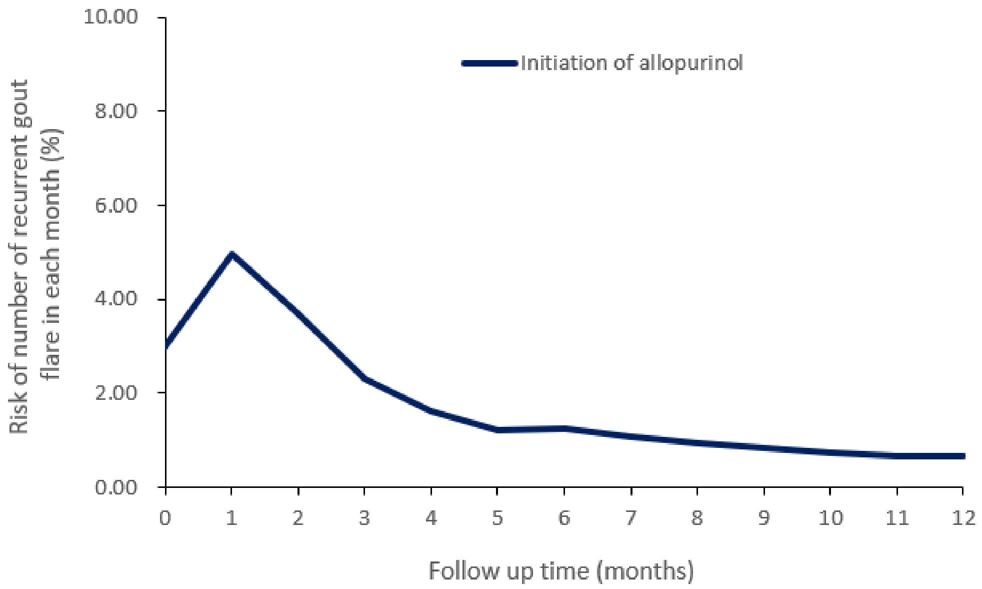

© 2023 Wei J et al. *JAMA Network Open*.

eFigure 5. Cumulative Incidence of All-Cause Mortality After Initiation of Either SGLT2i or Active Comparators

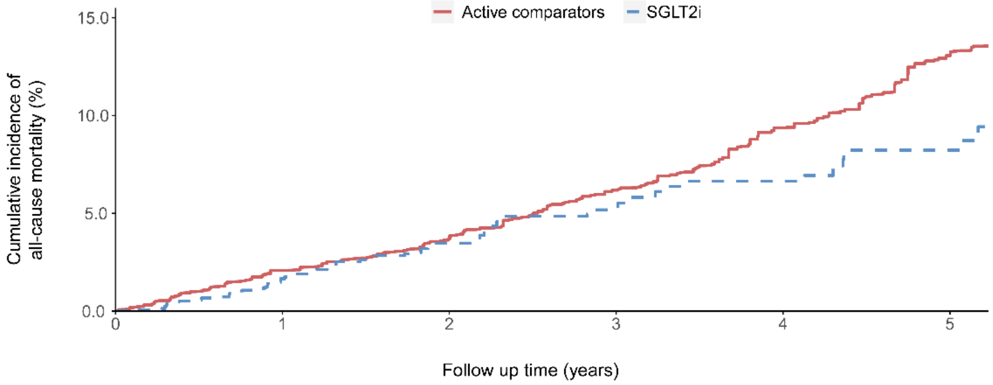

No. at risk						
SGLT2i	1548	1127	774	486	316	198
Active comparators	4383	3476	2667	1942	1423	996

SGLT2i, sodium glucose cotransporter-2 inhibitors. Active comparators consist of glucagon-like peptide-1 receptor agonist or dipeptidyl peptidase 4 inhibitors.

© 2023 Wei J et al. *JAMA Network Open*.
