# Supplementary material for: Gout Flares and Mortality After Sodium-Glucose Cotransporter-2 Inhibitor Treatment for Gout and Type 2 Diabetes
Source: JAMA Netw Open. 2023 Aug 25;6(8):e2330885. doi: 10.1001/jamanetworkopen.2023.30885 (PMC10457713; doi:10.1001/jamanetworkopen.2023.30885)
Supplement: Supplement 2. — Data Sharing Statement [file jamanetwopen-e2330885-s002.pdf]

Data Sharing Statement

Wei. Gout Flares and Mortality After Sodium-Glucose Cotransporter-2 Inhibitor Treatment for Gout and Type 2 Diabetes. *JAMA Netw Open*. Published August 25, 2023.
doi:10.1001/jamanetworkopen.2023.30885

Data
Data available: No

Additional Information
Explanation for why data not available: Data is available for purchase from info@the-health-improvement-network.co.uk
